# Mailed versus frozen transport of nasal swabs for surveillance of respiratory bacteria in remote Indigenous communities in Australia

**DOI:** 10.1186/1471-2334-13-543

**Published:** 2013-11-14

**Authors:** Kerry-Ann F O’Grady, David M Whiley, Paul J Torzillo, Theo P Sloots, Stephen B Lambert

**Affiliations:** 1 Queensland Children’s Medical Research Institute, Queensland University of Technology, Herston Road HERSTON QLD, 4029 Herston, Australia; 2 Queensland Paediatric Infectious Diseases Laboratory, Queensland Children’s Medical Research Institute, The University of Queensland, Herston, Australia; 3 Sydney Medical School, University of Sydney, Sydney, Australia; 4 Nganampa Health Service, Anangu Pitjantjatjara Yankunytjatjara, Alice Springs, Australia; 5 Sir Albert Sakzewski Virus Research Centre, Royal Children’s Hospital, Herston, Australia; 6 Queensland Children’s Medical Research Institute, The University of Queensland, Herston, Australia; 7 Communicable Diseases Branch, Queensland Health, Brisbane, Australia

**Keywords:** Respiratory bacteria, RT-PCR, Specimen transport, Laboratory methods

## Abstract

**Background:**

Surveillance programs and research for acute respiratory infections in remote Australian communities are complicated by difficulties in the storage and transport of frozen samples to urban laboratories for testing. This study assessed the sensitivity of a simple method for transporting nasal swabs from a remote setting for bacterial polymerase chain reaction (PCR) testing.

**Methods:**

We sampled every individual who presented to a remote community clinic over a three week period in August at a time of low influenza and no respiratory syncytial virus activity. Two anterior nasal swabs were collected from each participant. The left nare specimen was mailed to the laboratory via routine postal services. The right nare specimen was transported frozen. Testing for six bacterial species was undertaken using real-time PCR.

**Results:**

One hundred and forty participants were enrolled who contributed 150 study visits and paired specimens for testing. Respiratory illnesses accounted for 10% of the reasons for presentation. Bacteria were identified in 117 (78%) presentations for 110 (79.4%) individuals; *Streptococcus pneumoniae* and *Haemophilus influenzae* were the most common (each identified in 58% of episodes). The overall sensitivity for any bacterium detected in mailed specimens was 82.2% (95% CI 73.6, 88.1) compared to 94.8% (95% CI 89.4, 98.1) for frozen specimens. The sensitivity of the two methods varied by species identified.

**Conclusion:**

The mailing of unfrozen nasal specimens from remote communities appears to influence the utility of the specimen for bacterial studies, with a loss in sensitivity for the detection of any species overall. Further studies are needed to confirm our finding and to investigate the possible mechanisms of effect.

**Clinical trial registration:**

Australia and New Zealand Clinical Trials Registry Number: ACTRN12609001006235.

## Background

The rates of acute and chronic infections of the upper and lower respiratory tract in Indigenous children in remote communities in Australia are amongst the highest reported worldwide [1,2]. Furthermore, repeated infections in childhood are thought to contribute to the high rates of chronic lung disease in both adolescents and adults in these communities [3].

Despite the excess burden of disease, with the exception of ear disease, [4,5] there are no studies that have addressed the aetiology and epidemiology of non-hospitalised respiratory symptoms and acute respiratory illnesses in both adults and children at the community level in remote areas. One of the major impediments to this work has been the limited capacity for the collection, handling, and transport of respiratory specimens. There is a critical need for studies to both better understand respiratory disease epidemiology and to evaluate the efficacy of interventions, such as vaccines.

We undertook a study to assess the sensitivity of a simple, cost-efficient method for transporting nasal swabs from a remote setting for viral and bacterial real-time PCR compared with transport using frozen specimens. Our primary hypothesis was that there was no difference in the proportion of organisms detected between mailed and frozen discordant pairs. We have previously reported our findings with respect to viruses [6]. Here we report the findings of the bacterial component of the study.

## Methods

The study was conducted in a remote desert community in Central Australia over a period of three weeks from late August to mid-September 2009. The community is approximately 5 hours drive from the nearest regional centre, predominantly via unsealed, rough roads. It has an estimated resident population of 580, although this fluctuates throughout the year. Daily presentation at the community clinic ranges from 20 to 50 people. Data from our viral studies indicated low influenza and no RSV activity [6].

### Study participants

We convenience sampled any person presenting to the local health clinic who had spent the majority of the previous 14 days in the community. Individuals could either be presenting with a medical complaint, visiting, or accompanying a friend or family member. Individuals could participate in the study multiple times during the course of the three weeks.

### Data collection

We collected basic demographic and clinical data from enrolled subjects including: Indigenous status, reason for presentation at the clinic, clinic diagnosis for those seen by clinic staff, current respiratory and general symptoms, medications taken within the previous 7 days, and vaccination history.

### Clinical specimens

Sample collection and viral testing are previously described [6]. Briefly, two anterior nose swabs (from the nasal vestibule just inside the nares), one from each nostril, were collected using the Virocult collection system (MW950, Medical Wire & Equipment, England). Swabs taken from the left nostril were stored in the clinic refrigerator prior to being transported by normal post (air transport) at ambient temperature from the community once a week as exempt human specimens in accordance with Australia Post’s Dangerous and Prohibited Goods and Packaging requirements [7]. The right nare swab specimen was stored immediately at −20°C in a portable freezer. At the end of the data collection period, they were transported in that freezer to the Menzies School of Health Research laboratory in Darwin, Northern Territory and stored at −80°C. Frozen swabs were subsequently transported overnight to the Queensland Paediatric Infectious Diseases Laboratory in Brisbane, Queensland, in a dry ice shipper (with continuous electronic temperature monitoring) where they were again stored at −80°C until analysis occurred. At the laboratory, prior to extraction, 2 ml of VTM was added to the swab transport tube and thoroughly mixed by a vortex. Each sample was extracted using the Qiagen X-tractor Gene instrument (Qiagen, Australia) and the Qiagen DX reagents kit (Qiagen) according to manufacturer’s instructions.

Specimen extracts were tested by real-time PCR for the presence of *Bordetella pertussis*, *Haemophilus influenzae, Moraxella catarrhalis, Streptococcus pneumoniae, Staphylococcus aureus,* and methicillin-resistant *Staphylococcus aureus* (MRSA). Standardised reaction mix and cycling conditions were used; each reaction mix consisted of 12.5 μl of QuantiTect Probe PCR Master Mix, (QIAGEN, Doncaster, Australia); 10.0 pmol of forward and reverse primers (Table 1), 4.0 pmol of probe (Table 1) and 5.0 μl of sample nucleic acid extract in a final reaction volume of 25.0 μl. Reactions were cycled on an ABI7500 real-time PCR instrument (Applied Biosystems, USA) with the following parameters: initial hold at 95°C for 15 min followed by 45 cycles at 95°C for 15 seconds and 60°C for 60 seconds. Results were analysed using amplification plot data and were considered positive if amplification curves crossed above background fluorescence. Given previously described specificity problems associated with *H. influenzae* PCR methods; [8] specimens needed to be positive by two *H. influenzae PCR targets* (*hpd3* and *p6*; Table 1) to be considered positive for *H. influenzae.* MRSA was differentiated from *S. aureus* as described by Renwick et al. [9]. As previously described, we assessed the quality of specimen collection by evaluating the presence of marker of human genomic DNA, ERV3 [6].

**Table 1:** List of primers and probes used in the study

**Bacteria**	**Designation**	**Oligonucleotide sequence**	**Gene**	**Reference**
*Streptococcus pneumoniae*	*F primer*	AGCGATAGCTTTCTCCAAGTG	*ply*	[10]
	*R primer*	CTTAGCCAAATCGTTTACCG		
	*Probe*	FAM*-ACCCCAGCAATTCAAGTGTTCGCG- BHQ1^**§**^		
*Moraxella catarrhalis*	*F primer*	GTGAGTGCCGCTTTTACAA	*copB*	[11]
	*R primer*	CCGTGAGTGCCGCTTTTACAACC		
	*Probe*	FAM –TGCTTTTGCAGCTGTTAGCCAGCCTAA- BHQ1		
*Haemophilus influenzae*	*F primer*	GGTTAAATATGCCGATGGTGTTG	*hpd3*	[12]
	*R primer*	TGCATCTTTACGCACGGTGTA		
	*Probe*			
*Haemophilus influenza*	*F primer*	CCAGCTGCTAAAGTATTAGTAGAAG	*p6*	[13]
	*R primer*	TTCACCGTAAGATACTGTGCC		
	*Probe*	FAM- CAGATGCAGTTGAAGGTTATTTAG–BHQ1		
*Staphylococcus aureus*	*Staphylococcus aureus*	GTTGCTTAGTGTTAACTTTAGTTGTA	*nuc*	[14]
	*Staphylococcus aureus*	AATGTCGCAGGTTCTTTATGTAATTT		
	*Probe*	FAM- AAGTCTAAGTAGCTCAGCAAATGCA–BHQ1		
*MRSA*^*Ŧ*^	*mec2-F*	*mec2-F*	*SCCmec*	[9]
	*mec3-F*	ATTTCATATATGTAATTCCTCCACATCTC		
	*mec4-F*	CAAATATTATCTCGTAATTTACCTTGTTC		
	*mec5*-F	CTCTGCTTTATATTATAAAATTACGGCTG		
	*mec7*-F	CACTTTTTATTCTTCAAAGATTTGAGC		
	*orfX-R*	GGATCAAACGGCCTGCACA		
	*Probe*	FAM-CRTAGTTACTRCGTTGTAAGACGTC-BHQ1		
*Bordetella pertussis*	*F primer*	ATCAAGCACCGCTTTACCC	*IS 481*	[15]
	*R primer*	TTGGGAGTTCTGGTAGGTGTG		
	*Probe*	FAM- AATGGCAAGGCCGAACGCTTCA–BHQ1		

*FAM = 6-Carboxyfluorescein ^**§**^BHQ1 = Black Hole Quencher-1 ^*Ŧ*^MRSA = Methicillin resistant *S. aureus.*

### Data analysis

We performed descriptive analyses of demographic and clinical data, which are presented as proportions of all participants or all paired specimens collected. Given the sensitivity and specificity of real-time PCR diagnosis, we considered a specimen from either nostril positive for any bacterial species to represent a true-positive. This approach means that the specificity of either specimen type for any bacterial species will be, by definition, 100%. Using this standard, we calculated sensitivity overall and by specific species with 95% confidence intervals (CIs). Binomial probability was used to test our primary hypothesis of no difference in the likelihood of bacterial species detection in either the mailed or frozen sample for discordant specimens, with α = 0.05 (two-sided test).

Cycle threshold (Ct) values (cycle number at the threshold level of log-based fluorescence) are a semi-quantitative marker of, and are indirectly proportional to, nucleic acid load. A 3.3 cycle difference represents approximately a one-log difference in nucleic acid load [16]. Specimens with a cycle threshold count of less than 40 for individual species were considered positive. To investigate the influence of low DNA loads on the discordant results observed between the paired samples, we reanalysed the data by excluding low DNA loads. For this analysis, samples providing cycle threshold values of 36 cycles or above in the real-time PCR were deemed to contain low DNA loads and were reassigned as negative. All data were analysed using Stata 11 for Windows (Stata Corp, College Station, TX).

### Ethical conduct

We obtained signed informed consent from adult participants, assent from 10 to 18 year olds, and parent or guardian consent for children up to 18 years of age. The project was approved by the Central Australia Health Research Ethics Committee, which includes an Indigenous research ethics subcommittee, and by the Board of the region’s Aboriginal Community Controlled Health Service.

## Results

Over the three weeks of the study, we recruited 140 participants (82% of those screened) who contributed 153 study visits. Three study visits were excluded from further analysis due to inadequate specimen identification, leaving 150 paired specimens for analysis. There were no temperature deviations during storage of frozen specimens (maximum temperature −5°C) and the range for mailed specimens during transport was 12.8 to 30.5°C.

Eighty-two percent of the participants were Aboriginal, with the remainder being Caucasian, 60% were female and 21% were aged less than five years. Eight individuals were seen twice and three were seen three times. The reason for presentation at the clinic was for an acute upper respiratory illness in 10 (6.7%) visits, acute lower respiratory illness in 3 (2.0%) visits, chronic respiratory illness in 2 visits (1.4%), other medical conditions in 43 (28.7%) and visiting or accompanying another person in 92 (61.3%) visits [6].

At least 1 bacterial species was identified in 117 (78%) presentations for 110 (79.4%) individuals. In the 117 episodes where any bacterial species was identified, 23 had only one species identified (19.6%), 28 (23.9%) had two species, and 66 (56.4%) had three or more species detected. There were 64 (54.7%) specimen pairs that were discordant for at least one species; 18 were positive only in the mailed specimen and 46 were positive only in the frozen specimen (p = 0.006). There was concordance between paired specimens for at least one species in 90 episodes (76.9%); amongst these there were 39 (42.4%) episodes in which discordance between specimen pairs for a different species was also present. The overall sensitivity for any species detected in mailed specimens was 82.2% (95% CI 73.6, 88.1) compared to 94.8% (95% CI 89.4, 98.1) for frozen specimens (Table 2). The sensitivity of the two methods varied by species identified (Table 2).

**Table 2:** Sensitivity for the detection of different bacteria according to specimen transport method and by cycle threshold cut-off value

	**Cycle threshold cut off ≤40**					**Cycle threshold cut off ≤36**				
**Bacterium**		**Mailed (Left nare)**		**Frozen (Right nare)**			**Mailed (Left nare)**		**Frozen (Right nare)**	
	**Episodes +ve**	**Sensitivity%**	**95% CI**	**Sensitivity%**	**95% CI**	**Episodes +ve**	**Sensitivity%**	**95% CI**	**Sensitivity%**	**95% CI**
*S. pneumoniae*	87	78.2	68.0 – 86.3	97.7	92.0 – 99.7	72	84.7	74.3 – 92.1	93.1	84.5 – 97.7
*H. influenzae*	87	69.0	58.1 – 78.5	97.7	91.9 – 99.7	66	77.3	65.3 – 86.7	95.5	87.3 – 99.1
*S. aureus*	29	82.7	64.2 – 94.2	82.7	64.2 – 94.2	17	64.7	38.3 – 85.8	76.5	50.1 – 93.2
*MRSA*	8	75	34.9 – 96.8	62.5	24.5 – 91.5	3	0	-	100	-
*B. pertussis*	3	66.7	9.4 – 99.2	33.3	0.8 – 90.6	0	-	-	-	-
*M. catarrhalis*	77	80.5	69.9 – 88.7	97.4	90.3 – 99.7	63	82.5	70.9 – 90.9	93.7	84.5 – 98.2
*Any species*	***117***	*82.2%*	*73.6, 88.1*	*94.8%*	*89.4, 98.1*	*96*	*85.4*	*76.7 – 91.7*	*93.8*	*86.9 – 97.7*

ERV3 Ct values were higher (lower nucleic acid load) in mailed specimens (mean: 33.1, 95% CI 33.0, 33.2) compared to frozen specimens (mean: 31.5, 95%CI 31.4, 31.6); absolute difference 1.6 (95% CI 1.5, 1.7). The mean ERV3 Ct value in episodes that were bacteria positive in the left nare only was 32.5 (95% CI 29.2, 35.8) and the mean for episodes that were positive in the right nare only was 31.8 (95% CI 30.5, 33.2); absolute difference 0.7 (95% CI -4.5, 1.5).

After excluding specimens with low DNA loads, a significant improvement (p < 0.0001) was observed in paired sample result concordance, with 97 sample pairs proving concordant. As might be expected, the overall number of episodes that were positive for any species declined; there was only marginal improvement in sensitivity for mailed specimens (Table 2) and, conversely, a decline in sensitivity for *S. aureus*.

## Discussion

Studies aimed at investigating respiratory illness and the microbiology of those illnesses in remote communities have to date been limited by the inability to store and transport clinical specimens requiring freezing/refrigeration to urban laboratories. Our study has demonstrated that freezing of specimens for viral studies at the time of collection is not required, [6] however the detection of common respiratory bacteria is affected, with lower detection rates in mailed specimens.

The reasons for our findings are not clear, particularly given similar divergence was not identified for viruses [6]. Overall, it is evident that there is at least a moderate loss in respiratory bacteria PCR sensitivity using the mailed samples compared to those that were transported frozen. This is further supported by the fact that the human DNA concentrations in the mailed samples were significantly lower (as indicated by ERV3 PCR) in the mailed samples, suggesting some DNA loss, possibly by degradation, in the posted samples. However, the amount of DNA loss that was observed (ie. to increase ERV3 Ct values by 1.6 cycles) cannot fully explain the lack of concordance between the paired samples, particularly the fact that some individuals had a very high load of a particular bacterial species in one nostril, but were negative for the same species in the other nostril. Focal bacterial colonies may be present in specific individuals but these would be expected to be randomly distributed between the left and right nostrils in the population as a whole. Furthermore for focal colonies to be a plausible explanation for our finding of higher sensitivity in frozen swabs (right nare swabs), this would need to assume that bacteria were more likely to colonise the right rather than the left nare. There are no data to support this assumption and we are currently testing this assumption by evaluating transport methods for swabs from taken from the same nare. Alternatively, given the overlap in confidence intervals for the sensitivities for each organism by transport method (Table 2), our findings may be a factor of inadequate sample size to compare differences in common respiratory bacteria.

We were unable to find similar studies in the literature with which to compare our findings. Our study was based on the assumption that there would be minimal, if any, differences in presence and load of viral and bacterial pathogens in the left and right nostrils of individuals, and appeared valid on the basis of our findings with respect to viruses. However, we now postulate that this is not that case and that higher concordance between transport methods may have been observed for the bacterial pathogens had two swabs from the same nostril been used. As above, a further study is in progress to address this issue.

Our findings need to be considered in light of the feasibility and cost of respiratory pathogen surveillance in remote areas where compromises in sensitivity may be required in order for research to occur [17]. Overall sensitivities for any bacteria detected were acceptable in that context, particularly when the collection of specimens for research purposes does not influence clinical management decisions at the time the specimen was collected. Furthermore, the ability to collect one specimen per participant that can be readily shipped and analysed for both viruses and bacteria is important given the increasing emphasis on virus-bacteria interaction in acute and chronic respiratory disease [18-21]. Ideally, nasal swabs are best stored frozen within a short-time frame at ultra low temperatures and shipping from a remote community using nitrogen shippers or dry ice shippers as the gold standard would be preferred. However, this is not logistically feasible in these settings, particularly when research staff are in the community for extended periods, there are limited facilities to maintain dry ice for these timespans and, air freight in and out of the community only occurs once a week. Similarly, future studies should consider the use of concurrent collection from both nares to maximise sampling efficiency.

Our choice of storage media (Virocult, MW950, Medical Wire & Equipment, England) was also based on logistics, cost and that bacterial culture would not be performed. The medium does not need to be stored refrigerated or frozen prior to use, unlike media commonly used for bacterial culture, and there is no risk of breakage during transport, an important consideration if postal services are used. Furthermore, there is no reason to believe that the media itself would affect PCR detection of bacterial DNA as indicated by our high overall positivity rates that are consistent with other studies in this population. It should also be noted that there have been some reports of false-positive results using the pneumococcal *ply* PCR used in this study, [22] and so the results here may represent a slight overestimation of carriage.

## Conclusions

Our study suggests that combining standard clinic refrigeration and weekly surface mailing of specimens combined with real-time PCR, is potentially useful for respiratory research in remote locations. However, further investigation of the difference in sensitivity detected between mailed and frozen specimens for common respiratory bacteria is warranted and currently planned. A simple, cost efficient approach is required to help us better understand the epidemiology of respiratory viruses and bacteria in remote, disadvantaged populations, where recurrent infections and persistent carriage are predominant causes of morbidity and mortality in children and may result in chronic respiratory disease.

## Competing interests

The authors declare that they have no competing interests.

## Author’s contributions

KO and SL designed the study, collected and analysed data and KO drafted the paper. PT contributed to the design and conduct of the study, including community consultation and assisted with obtaining study approval. DW and TS performed and/or assisted with laboratory analyses and interpretation of laboratory data. All authors contributed to and approved the final manuscript.

## Pre-publication history

The pre-publication history for this paper can be accessed here:

http://www.biomedcentral.com/1471-2334/13/543/prepub
